# Enhancing Heart Rate-Based Estimation of Energy Expenditure and Exercise Intensity in Patients Post Stroke

**DOI:** 10.3390/bioengineering11121250

**Published:** 2024-12-10

**Authors:** Anna Roto Cataldo, Jie Fei, Karen J. Hutchinson, Regina Sloutsky, Julie Starr, Stefano M. M. De Rossi, Louis N. Awad

**Affiliations:** Department of Physical Therapy, Sargent College of Health and Rehabilitation Sciences, Boston University, Boston, MA 02215, USA; aroto@bu.edu (A.R.C.); jfei@bu.edu (J.F.); kahutch@bu.edu (K.J.H.); reginas@bu.edu (R.S.); jstarr@bu.edu (J.S.); stefano@derossi.consulting (S.M.M.D.R.)

**Keywords:** volume of oxygen consumed, heart rate, subject-specific estimation, exercise intensity, relative VO_2_, stroke, motor training, physiological monitoring, rehabilitation

## Abstract

Background: Indirect calorimetry is the gold standard field-testing technique for measuring energy expenditure and exercise intensity based on the volume of oxygen consumed (VO_2_, mL O_2_/min). Although heart rate is often used as a proxy for VO_2_, heart rate-based estimates of VO_2_ may be inaccurate after stroke due to changes in the heart rate–VO_2_ relationship. Our objective was to evaluate in people post stroke the accuracy of using heart rate to estimate relative walking VO_2_ (wVO_2_) and classify exercise intensity. Moreover, we sought to determine if estimation accuracy could be improved by including clinical variables related to patients’ function and health in the estimation. Methods: Sixteen individuals post stroke completed treadmill walking exercises with concurrent indirect calorimetry and heart rate monitoring. Using 70% of the data, forward selection regression with repeated k-fold cross-validation was used to build wVO_2_ estimation equations that use heart rate alone and together with clinical variables available at the point-of-care (i.e., BMI, age, sex, and comfortable walking speed). The remaining 30% of the data were used to evaluate accuracy by comparing (1) the estimated and actual wVO_2_ measurements and (2) the exercise intensity classifications based on metabolic equivalents (METs) calculated using the estimated and actual wVO_2_ measurements. Results: Heart rate-based wVO_2_ estimates were inaccurate (MAE = 3.11 mL O_2_/kg/min) and unreliable (ICC = 0.68). Incorporating BMI, age, and sex in the estimation resulted in improvements in accuracy (MAE Δ: −36.01%, MAE = 1.99 mL O_2_/kg/min) and reliability (ICC Δ: +20, ICC = 0.88). Improved exercise intensity classifications were also observed, with higher accuracy (Δ: +29.85%, from 0.67 to 0.87), kappa (Δ: +108.33%, from 0.36 to 0.75), sensitivity (Δ: +30.43%, from 0.46 to 0.60), and specificity (Δ: +17.95%, from 0.78 to 0.92). Conclusions: In people post stroke, heart rate-based wVO_2_ estimations are inaccurate but can be substantially improved by incorporating clinical variables readily available at the point of care.

## 1. Introduction

Stroke is a leading cause of long-term disability [1], with stroke-induced neuromotor impairments significantly impacting daily life activities [2,3]. Reduced physical activity post stroke contributes to a vicious cycle of cardiovascular and muscular deconditioning, loss of functional autonomy, and increased risk of secondary stroke [4,5]. The recovery of motor function depends heavily on neuroplasticity mechanisms such as synaptic plasticity and axonal sprouting [6,7,8]. Rehabilitation strategies, including gait training and other task-specific interventions, use repetitive movement practice to induce the neuroplastic changes necessary for motor relearning and functional recovery [9,10]. Advancing therapies that enhance exercise-induced neuroplasticity is both a clinical [11,12] and scientific priority.

Exercise dosage, specifically the amount and intensity of task-specific training, has emerged as a critical determinant of the motor recovery during post-stroke rehabilitation [13,14]. Exercise intensity, defined as the rate of work or power, is typically quantified by measuring the energy required to perform a given activity [15]. For gait training, energy demands are predominantly met through aerobic (oxygen-dependent) metabolism, making the volume of oxygen consumed (VO_2_, mL O_2_/min) the gold standard for measuring exercise intensity [16,17]. Beyond promoting cardiopulmonary adaptations [18,19,20], exercise intensity is also recognized for its role in signaling the neuroplastic mechanisms underlying motor learning [21,22,23,24,25,26,27,28]. Consequently, recent clinical practice guidelines [12,29] highlight the importance of prescribing exercise intensity to promote both cardiovascular health [30] and the neuroplastic changes essential for motor recovery [12,25].

Relative VO_2_, the volume of oxygen consumed relative to body weight (mL O_2_/kg/min), is widely used to determine if an exercise activity is of sufficient intensity to elicit physiological adaptations. A common method for classifying exercise intensity based on relative VO_2_ uses metabolic equivalents (METs) [31], where one MET corresponds to resting oxygen consumption (i.e., ~3.5 mL O_2_/kg/min). Moderate intensity is defined as between 3.0 and 5.9 METs, and vigorous intensity as 6.0 + METs [32]. While laboratory-based techniques such as indirect calorimetry provide accurate and real-time monitoring of relative VO_2_, these methods require expensive equipment and specialized expertise [33], making them impractical for routine clinical use. As a result, clinicians often rely on heart rate as a proxy for VO_2_.

The relationship between heart rate and VO_2_ underpins its use as a proxy [34]. Indeed, exercise-induced increases in oxygen demand are typically met by a rise in myocardial contractility, driven primarily by increases in heart rate [35]. Increased heart rate, together with an increase in stroke volume, contribute to the increase in cardiac output during exercise that ultimately supplies oxygenated blood to the working muscles [35,36]. However, several factors influence the relationship between heart rate and VO_2_ [5,37,38]. For example, time of day [39], food and caffeine intake, dehydration, medications, ambient temperature, and humidity [36] each affect the heart rate–VO_2_ relationship. Consequently, heart rate-based VO_2_ estimation can incur errors of up to 25% [39,40]. Stroke-related physiological changes can also disrupt the heart rate–VO_2_ relationship. Specifically, the impaired capacity of skeletal muscle to extract and utilize oxygen from circulation [37,38], along with autonomic dysfunction [5], may blunt or delay heart rate responses to exercise, while vascular changes can impair peripheral oxygen delivery and utilization [41]. These alterations make VO_2_ difficult to accurately predict from heart rate and necessitate the development of new approaches for estimating energy expenditure in the post-stroke population.

Given the importance of accurately measuring exercise intensity during neurorehabilitation and the limitations of using heart rate alone to estimate VO_2_ and exercise intensity, there is a need for VO_2_ estimation methods that combine the usability of heart rate monitoring with the accuracy of indirect calorimetry. The objective of this study was to develop and validate a VO_2_ estimation equation that incorporates clinical variables available at the point-of-care to enhance the accuracy of heart rate-based estimates. We hypothesized that clinical variables such as BMI, age, sex, and comfortable walking speed—which are both easy to collect in most clinical settings and are widely used when describing the functional and health status of the individual post stroke—would improve VO_2_ estimation accuracy and reliability. Furthermore, we applied the VO_2_ estimates to classify exercise intensity based on METs and compared the classifications to those derived from indirect calorimetry measurements.

## 2. Methods

### 2.1. Participants

In this study, the participant data from two different experimental studies, each enrolling different participant cohorts, were combined to enable the development and validation of a robust VO_2_ estimation equation. Cohort 1 included eleven community-dwelling individuals with chronic stroke who completed symptom-limited maximal effort graded exercise testing. Cohort 2 included five community-dwelling individuals with chronic stroke who completed bouts of treadmill walking at different speeds [42]. Study inclusion criteria for both cohorts were similar and included being more than six months after stroke, ambulatory but with residual gait deficits, and having the ability to walk on a treadmill without orthotic support. Study exclusion criteria for both cohorts were similar and included having a cerebellar stroke, lower extremity joint replacement or other orthopedic conditions that affect walking ability, taking heart rate altering medications (i.e., beta-blockers), pain that limits walking ability, inability to communicate with investigators, neglect or hemianopia, unexplained dizziness, or more than two falls in the previous month. All study procedures were approved by the Institutional Review Boards overseeing each study. Written informed consent was obtained for all participants.

### 2.2. Data Collection and Processing

For both cohorts, heart rate and VO_2_ data were collected concurrently. Data from both cohorts were pooled and then split so that 70% of the data were used for equation development, while the remaining 30% were reserved for validation testing.

#### 2.2.1. Cohort 1 Data Collection: Graded Exercise Testing

Participants first completed a clinical and physical screen with a licensed physical therapist to assess study eligibility, characterize stroke severity, and collect demographic and anthropometric information. Participants also completed 10 m walk tests to measure walking speed. A supine resting 12-lead electrocardiography (Quark C12x ECG; Cosmed, Rome, Italy) recording was taken to assess resting cardiovascular characteristics and rule out the presence of any abnormal heart rhythm prior to exercise.

To begin the graded exercise test, participants were instructed to stand quietly for 5 min while continuous indirect calorimetry and heart rate data were collected on a breath-by-breath basis (K5; Cosmed, Rome, Italy). Following the standardized rest capture, participants performed either a speed- or incline-based graded exercise test. Because post-stroke neuromotor impairment is heterogenous and differentially limits an individual’s ability to walk faster or on an inclined surface, including both speed- and incline-based tests allowed for a greater range of aerobic demands than may be achieved by one mode of testing alone. During speed-based tests, participants walked on a treadmill at their comfortable walking speed for two minutes, followed by a 10% increase in treadmill speed every two minutes until test termination [43]. During incline-based tests, participants walked on a treadmill at 85% of their fastest overground walking speed, while the treadmill incline was increased by 2% or 4% every two minutes until test termination [44].

Test duration varied due to the nature of the self-limited test coupled with the variability in physical abilities after stroke, with an average of 14.5 ± 4 min (ranging from 6 to 22 min). Stopping criteria for both tests included reaching peak volitional fatigue or meeting any of the physical or physiological stopping criteria [36,44,45,46]. Participants were connected to an overhead harness for safety purposes only; the harness did not provide any body weight support.

#### 2.2.2. Cohort 2 Data Collection: Treadmill Walking at Different Speeds

Detailed descriptions of study procedures completed by Cohort 2 have been previously described [42]. In brief, all participants completed two testing visits. The first consisted of a standardized clinical evaluation during which demographic and anthropometric information was collected, stroke severity (i.e., FGA score) was characterized, and gait tests were performed (see Table 1). The second testing visit began with a 4 min seated rest period to collect resting indirect calorimetry and heart rate data, sampled at 30 s intervals (Oxycon Mobile, Yorba Linda, CA, USA). Participants then completed a brief acclimation and warm-up period on the treadmill, followed by four separate trials of three-minute treadmill walks completed in random order:

i. Slow speed: 20% slower than comfortable 10 m walk test walking speed;
ii. Comfortable walking speed: comfortable 10 m walk test walking speed;
iii. Fast 1 speed: the average 6 min walk test speed;
iv. Fast 2 speed: maximum 10 m walk test walking speed.

#### 2.2.3. Data Processing

For both cohorts, the VO_2_ data set was time-sliced to include only oxygen consumption data during walking (i.e., wVO_2_) collected during an energetic steady state, defined by a standard deviation less than or equal to 2.0 mL O_2_/kg/min [47]. wVO_2_ data outliers were defined as breaths greater than two standard deviations from the average wVO_2_ and removed. Steady-state wVO_2_ data were normalized by body weight (kg) to yield the relative wVO_2_ (hereinafter, relative wVO_2_ is referred to as wVO_2_). The wVO_2_ achieved by participants during testing is reported in the Supplementary Materials (Table S1).

### 2.3. Analyses

wVO_2_ estimation equations were developed by automated forward selection regression with repeated k-fold cross-validation (10-fold, repeated three times) using 70% of the pooled data. The remaining 30% of data were reserved for validation, which consisted of comparing the accuracy of equation-generated wVO_2_ estimations to actual wVO_2_ measurements by the gold-standard, indirect calorimetry. All analyses were performed in RStudio Version 1.3.1056. Alpha was set to 0.05.

#### 2.3.1. Equation Development

The repeated k-fold cross-validation resampling method randomly split the testing data set into k = 10 subsets of approximately equal size allowing the entire data set to be shuffled and split into different data subsets for equation development. Forward selection regression began with an equation that contains no variables (only an intercept) and added candidate predictor variables in a stepwise manner based on predictive significance, thus resulting in the best 1-variable, 2-variable, etc., equation(s). The candidate predictor variables considered during equation development were heart rate, body mass index (BMI), age, sex, and comfortable walking speed (CWS). The criteria for including candidate predictors were the following: (1) previous research indicating a relationship between the candidate predictor and energy expenditure in humans [48,49,50,51]; (2) ease of collection at the point-of-care; and (3) availability for all research participants. Criteria for excluding candidate predictors were the following: (1) previous research does not clearly indicate a relationship between the candidate predictor and energy expenditure; (2) not easily collected at the point-of-care (e.g., uptake and utilization of oxygen by the muscles during walking/arteriolar-venous oxygen difference); and/or (3) not available for all participants (e.g., stroke chronicity and fast walking speed measured by the 10 m walk test). Equation fit was assessed using common variable selection summary metrics of adjusted R^2^, Mallow’s C(p), Akaike information criterion (AIC), Sawa’s Bayesian information criterion (SBIC), average prediction errors of mean absolute error (MAE), and root mean square error (RMSE). A higher adjusted R^2^ and lower C(p), AIC, SBIC, and lower MAE and RMSE values indicate better fit and wVO_2_ estimation accuracy, respectively. A meaningful improvement was defined in this study as a change greater than 5% in RMSE or MAE.

#### 2.3.2. Equation Validation

Utilizing 30% of the data set reserved for validation testing, and not used during any part of equation development, the actual wVO_2_ measurements collected by the gold-standard method, indirect calorimetry, were compared to wVO_2_ estimations generated using each estimation equation produced during equation development (i.e., the best 1-variable equation, best 2-variable equation, etc.). Estimation accuracy was quantified using R^2^, MAE, and RMSE, with larger R^2^ and smaller MAE and RMSE values reflecting a better fit and higher estimation accuracy. Estimation reliability was assessed using two-way mixed effects, absolute agreement, and single rater intra-class correlation coefficients (ICC). ICC values above 0.90 were considered to reflect excellent reliability, 0.90 to 0.75 as good reliability, 0.75 to 0.50 as moderate reliability, and less than 0.50 as poor reliability [52]. To determine whether the addition of candidate predictor variables had a meaningful effect on the equation’s performance, we reported percent changes in RMSE and MAE in a stepwise manner between each estimation equation produced during equation development. A meaningful improvement was defined in this study as a change greater than 5% in RMSE or MAE.

#### 2.3.3. Application: Classification of Exercise Intensity

Exercise intensity was classified using metabolic equivalents (METs) following the American College of Sports Medicine (ACSM) Exercise Intensity Guidelines. The actual and equation-estimated wVO_2_ were utilized to calculate METs based on formula as follows [31]. The value of 3.5 represents the typical metabolic equivalent of rest.

METs = wVO_2_/3.5

Exercise intensity was classified according to the ACSM exercise intensity guidelines as follows: (1) light intensity exercise: 1.6–2.9 METs; (2) moderate intensity exercise: 3.0–5.9 METs; (3) vigorous intensity exercise: 6.0 METs and above [32].

Additionally, the least accurate and reliable estimation equation, along with the most accurate and reliable equation with meaningful improvement, were utilized to calculate the METs and classify exercise intensity. The difference in classifying exercise intensity compared to actual exercise intensity between these two equations was quantified using confusion matrix, accuracy, kappa, sensitivity, and specificity. The confusion matrix describes the performance of classification using estimated METs (i.e., METs calculated from estimated wVO_2_) for which the actual METs (i.e., METs calculated from actual wVO_2_) values are known. Higher accuracy, kappa, sensitivity, and specificity values indicate better overall classification performance.

## 3. Results

The participants were 55.63 ± 11.85 years old (average ± standard deviation), 7.39 ± 5.08 years post stroke, 31.25% female, 80.55 ± 19.71 kg, and 173.99 ± 11.05 cm with a BMI of 26.46 ± 6.04 kg/m^2^. Their Functional Gait Assessment (FGA) score was 20.08 ± 4.98, with an FGA score not available for four participants (see Table 1). The average resting VO_2_ was 4.02 ± 1.07 mL O_2_/kg/min (see Supplementary Materials, Table S1).

### 3.1. Equation Development

**Figure 1:**
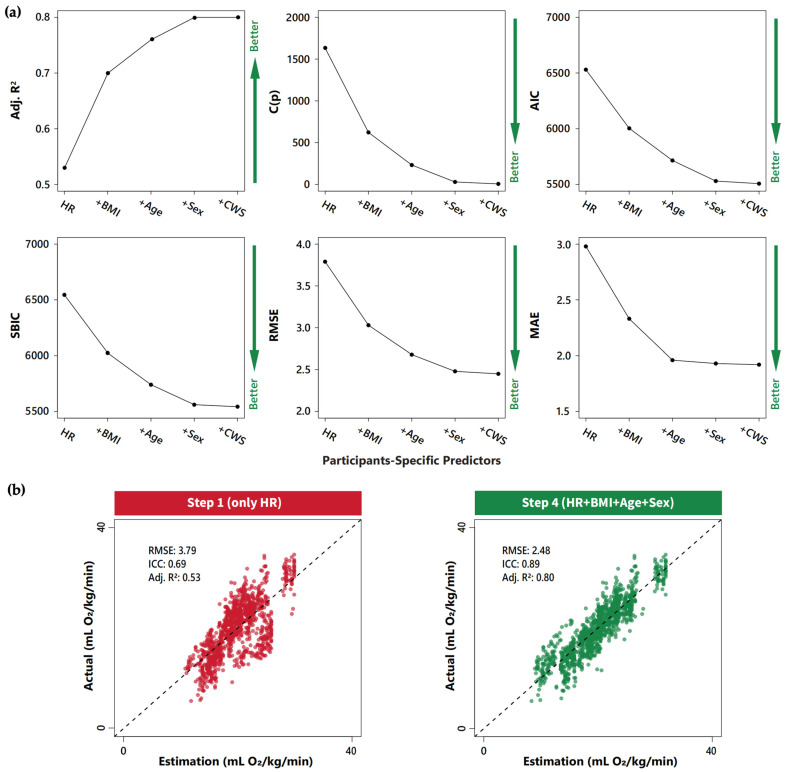
Stepwise improvements in wVO_2_ estimation from HR with the addition of participant-specific predictors: (**a**) equation fit and estimation accuracy per stepwise addition of predictors in automated forward selection regression. Equation fit and estimation accuracy were assessed using common predictor selection summary metrics of (in order of left to right) adjusted R-squared (Adj. R^2^), Mallow’s C(p), Akaike information criterion (AIC), Sawa’s Bayesian information criterion (SBIC), root mean square error (RMSE), and average prediction errors of mean absolute error (MAE). The direction of green arrows on the right side indicates the improvement for each metric: a higher adjusted R^2^ and lower C(p), AIC, SBIC, RMSE, and MAE values indicate better fit and wVO_2_ estimation accuracy, respectively; (**b**) scatter plot of actual versus estimated wVO_2_ for heart rate-only (Step 1) and best-performing estimation equations (Step 4). The left figure (in red) displays the accuracy of estimation using the heart rate-only equation (Step 1). The right figure (in green) displays the accuracy of estimation using the most accurate and reliable equation with meaningful improvement (Step 4, i.e., the fourth step of automated forward selection regression, incorporating heart rate, BMI, age, and sex as predictors).

Automated forward selection regression produced five estimation equations, ranging from the best 1- to 5-variable equation (see Figure 1a and Table 2). In the equation development data set, the estimated wVO_2_ moderately approximated the actual wVO_2_ when using the estimation equation based solely on heart rate (Step 1, RMSE = 3.79 mL O_2_/kg/min, MAE = 2.98 mL O_2_/kg/min, and Adj. R^2^ = 0.53). The addition of three predictors, BMI, age, and sex, meaningfully improved the overall fit and accuracy of the estimation (Step 4, RMSE = 2.48 mL O_2_/kg/min, MAE = 1.93 mL O_2_/kg/min, and Adj. R^2^ = 0.80) (see Figure 1 and Table 2). The last additional predictor, CWS, did not meaningfully improve the performance in estimating the wVO_2_ (%∆ RMSE = −1.21% and %∆ MAE = −0.52%).

### 3.2. Equation Validation

**Figure 2:**
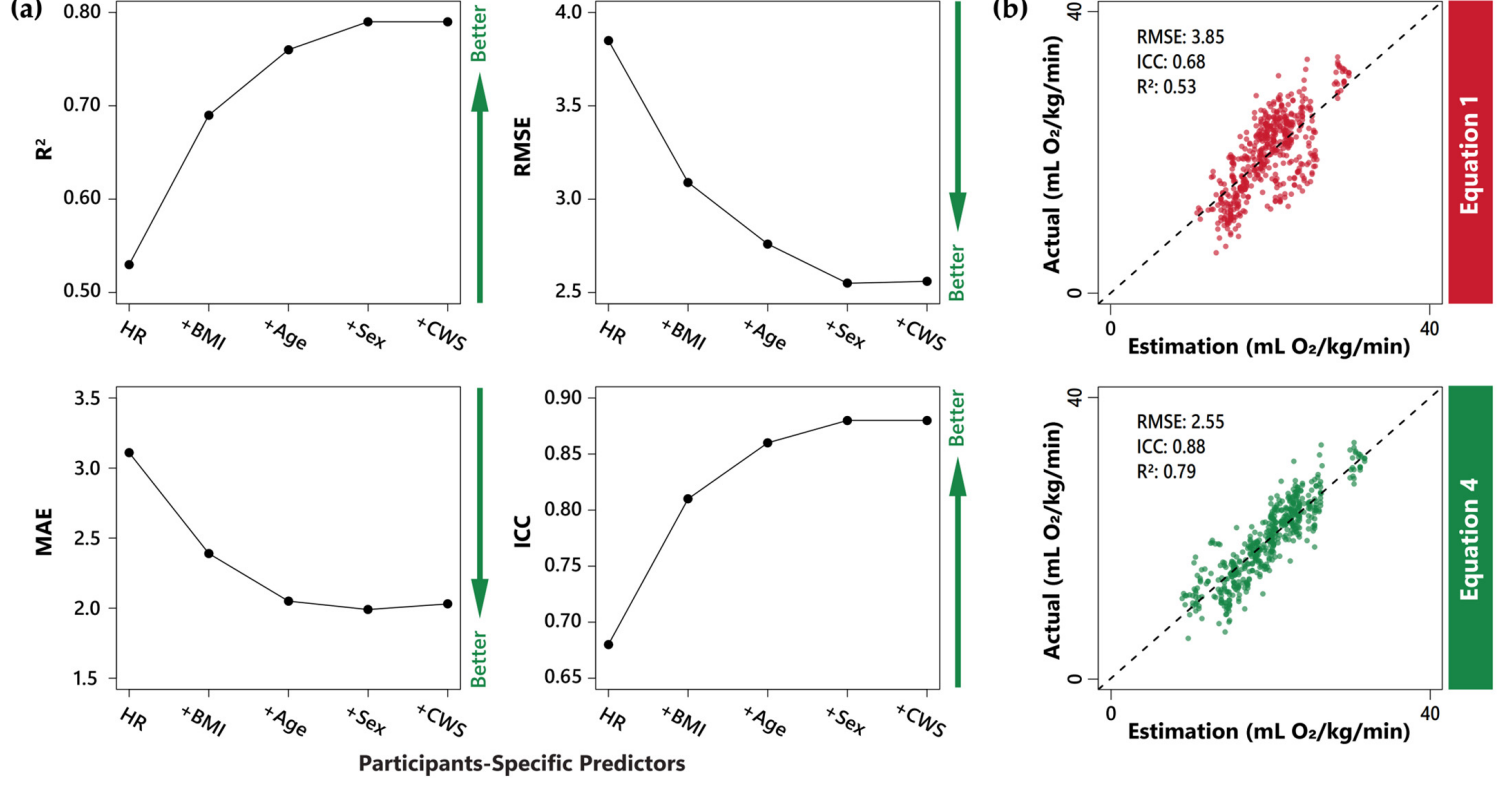
The performance of the equations in estimating wVO_2_ using validation data set: (**a**) the accuracy and reliability of different estimation equations. The equations’ accuracy, reliability, and goodness-of-fit were assessed using several metrics (listed from left to right), including R-squared (R^2^), root mean square error (RMSE), mean absolute error (MAE), and intra-class correlation coefficient (ICC). The direction of green arrows on the right side of each panel indicates the direction of improvement for each metric: higher R^2^ and ICC values, as well as lower RMSE and MAE values, signify better equation performance; (**b**) scatter plot of actual versus estimated wVO_2_ for Equations (1) and (4). The top figure (in red) shows the accuracy of estimation using Equation (1) with only heart rate as a predictor. The bottom figure (in green) displays the accuracy of estimation using the most accurate and reliable equation with meaningful improvement (i.e., Equation (4) with predictors including heart rate, BMI, age, and sex).

In the equation validation data set, wVO_2_ estimation made by the heart rate-only equation (Equation (1)) was the least accurate and reliable (RMSE = 3.85 mL O_2_/kg/min, ICC = 0.68, R^2^ = 0.53), see Figure 2 and Table 3. As additional predictors were added (Equations (2)–(5), see Table 3), the accuracy and reliability of the wVO_2_ estimates meaningfully improved up to Equation (4) (RMSE = 2.55 mL O_2_/kg/min, ICC = 0.88, and R^2^ = 0.79). Equation (4), along with its detailed coefficients, is reported below. The detailed coefficients for the other four equations are listed in Supplementary Table S2 for reference.

Estimated wVO_2_ (mL O_2_/kg/min) = 5.09 + 0.19 × HR (BPM) − 0.02 × BMI (kg/m^2^) − 0.18 × Age (y) + 4.53 × Sex (male = 0 and female = 1)

More specifically, in Equation (2), the addition of BMI to the estimation equation resulted in the largest stepwise reduction in RMSE and MAE (19.74% and 23.15%, respectively). In Equation (3), the addition of age resulted in a further reduction in RMSE and MAE (10.68% and 14.23%, respectively). In Equation (4), the addition of sex resulted in a further reduction in RMSE and MAE (7.61% and 2.93%, respectively). In Equation (5), the addition of CWS did not meaningfully improve the accuracy of the wVO_2_ estimates (i.e., less than a 5% change in RMSE or MAE), see Figure 2a and Table 3.

### 3.3. Application: Classification of Exercise Intensity Age, and Sex)

**Figure 3:**
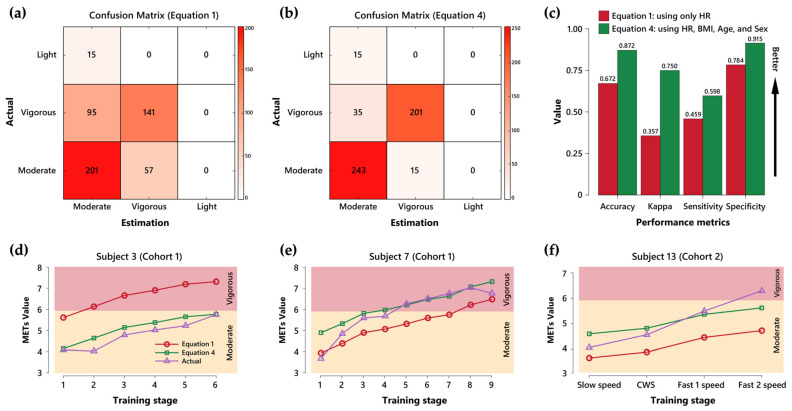
Comparison of performance between different estimation equations in classifying exercise intensity: (**a**) confusion matrix for Equation (1). Equation (1) uses only heart rate as the predictor. The 3 by 3 matrix shows the classification results for the Light, Moderate, and Vigorous categories. The *y*-axis represents actual categories, while the *x*-axis represents estimated categories. Each cell contains the number of instances classified, with a color scale from white to red indicating classification frequencies from low to high; (**b**) confusion matrix for Equation (4). Equation (4) uses heart rate, BMI, age, and sex as predictors. Similar to panel (**a**), this confusion matrix shows the classification results for Equation (4); (**c**) performance metrics comparison. A comparison of classification performance between Equations (1) and (4) evaluated using various metrics, including accuracy, kappa, sensitivity, and specificity. Red bars represent Equation (1), while green bars represent Equation (4). The *y*-axis ranges from 0 to 1, and the black arrow indicates better performance as values increase; (**d**) exercise intensity classification across training stages for subject 3. The *x*-axis represents increasing training stages from 1 to 6, while the *y*-axis shows average MET values. Red lines and circles indicate MET values estimated by Equation (1), green lines and squares indicate MET values estimated by Equation (4), and purple lines and triangles represent actual MET values. Different color zones denote exercise intensity classification per ASCM guidelines, with the red zone at the top representing vigorous intensity and the orange zone at the bottom representing moderate intensity; (**e**) exercise intensity classification for subject 7. The *x*-axis represents increasing training stages from 1 to 9; (**f**) exercise intensity classification for subject 13. The *x*-axis represents training stages ranging from slow speed to Fast 2 speed.

Equations (1) and (4) were used to calculate the estimated METs and classify the exercise intensity. Equation (4) generally outperforms Equation (1) across multiple metrics and subjects, see Figure 3. The confusion matrices in Figure 3a,b show Equation (4) has improved classification accuracy, particularly for the ‘Vigorous’ and ‘Moderate’ intensity categories. The bar chart in Figure 3c reveals that Equation (4) achieves better classification performance in accuracy (Δ: +29.85%, 0.87 vs. 0.67), kappa (Δ: +108.33%, 0.75 vs. 0.36), sensitivity (Δ: +30.43%, 0.60 vs. 0.46), and specificity (Δ: +17.95%, 0.92 vs. 0.78) compared to Equation (1). Additionally, the line graphs in Figure 3d–f, representing three sample subjects from the two cohorts, illustrate that Equation (4)’s MET value estimates (green line with squares) tend to align more closely with the actual MET values (purple lines with triangles) across different training stages, in contrast to Equation (1)’s MET value estimates (red line with circles).

## 4. Discussion

Recent studies and clinical practice guidelines for stroke gait rehabilitation have underscored the critical importance of prescribing and managing exercise intensity [3,53,54,55,56]. This emphasis serves the dual purpose of driving cardiovascular benefits and promoting the neuroplastic changes essential for motor learning and recovery. However, accurately measuring and monitoring exercise intensity in clinical settings presents significant challenges. The gold standard method for quantifying exercise intensity (i.e., indirect calorimetry) relies on sophisticated devices and expertise to measure VO_2_; its application in routine clinical practice is often impractical due to the high equipment costs and the specialized technical knowledge required for its operation [33]. The major finding of our study is that although wVO_2_ estimations made from heart rate alone are inaccurate, they can be substantially improved by incorporating readily available clinical variables related to function and health in the estimation equation. Critically, this enhanced estimation also enables a more accurate classification of exercise intensity.

The relationship between heart rate and VO_2_ is well documented [34,39]; however, lab-based methods using heart rate for exercise intensity monitoring have, historically, required individuals to complete a maximal effort-graded exercise test to plot a calibration curve describing the VO_2_–heart rate relationship for that individual [57]. This calibration curve is then used to predict VO_2_ consumption from heart rate during subsequent exercise activities on an individual basis, thus defining the individual’s metabolic intensity during exercise. While this approach provides accurate VO_2_ estimations from heart rate, it is generally unrealistic in non-laboratory settings where stroke rehabilitation typically takes place; it is time-consuming, requires a team of people with clinical and technical expertise, and puts the patient at a higher risk of adverse events (e.g., syncopal or cardiac events, physical injury, etc.) [58]. In contrast, the wVO_2_ estimation equation developed and validated in this study facilitates accurate and reliable wVO_2_ estimations using clinical variables collected easily at the point of care. That is, they do not require graded exercise testing to first define the individual VO_2_–heart rate relationship, and thus do not necessitate the same time commitment, expertise, and patient risk as traditional methods.

The enhanced wVO_2_ estimation equation reported in this study can be easily implemented at the point of care to track, prescribe, and monitor exercise intensity using more accurate and reliable estimates of wVO_2_ that do not require expensive laboratory devices. Compared to the heart rate-only equation, our best 4-variable estimation equation including heart rate, BMI, age, and sex (see Table 3, Equation (4)) improved estimation accuracy by more than a quarter (−34% RMSE and −36% MAE) and reliability by 20 points (+20 ICC). The accuracy of our 4-variable equation is similar to that of other energy estimation equations previously reported in chronic stroke [59,60,61] (R^2^ = 0.81 [61]) and neurologically intact subjects (mean difference between estimates and actual VO_2_ measures = 1.0–2.8 (males 51–60 years) and 1.4–3.3 mL O_2_/kg/min (females 51–60 years)) [62].

The candidate predictor variables examined in this study—i.e., BMI [63,64], age [36,65,66,67,68], sex [67], and comfortable walking speed [59,60,61]—were selected given their established influence on oxygen consumption in adults and the ease by which these data can be collected in clinic and community settings. In our data sample, BMI resulted in a marked reduction in wVO_2_ estimation error, the largest compared to any of the other variables considered. BMI serves as a general measure of adiposity [69], and, while not a specific metric of body composition, the measurement does attempt to capture information about body composition (by classifying the level of body fatness). Given body composition is a major factor in energy use [70], the fact that BMI describes aspects of body composition may suggest its potential for relating to VO_2_ [71]. Age and sex were the second and third predictor variables added by the automated forward regression procedure. Accurate VO_2_ estimation is essential for setting and monitoring energy expenditure and exercise intensity that align with rehabilitation goals, such as improving cardiovascular fitness, promoting neuroplasticity, and supporting functional independence. By incorporating variables like BMI, age, and sex, our estimation model offers clinicians a tool that facilitates tailored intensity prescriptions and progress tracking. To facilitate the clinical application of these findings, we provide (in Supplementary Materials S3) an Excel spreadsheet that automatically uses our optimal estimation equation to calculate estimated wVO_2_ and METs, and classify exercise intensity.

Though considered in our equation development process, estimation equations that added comfortable walking speed did not meaningfully improve the estimation accuracy (see Table 3, Equation (5)). This result contrasts with those of previously published energy estimation works in chronic stroke [59,60,61]. More specifically, Compagnat et al. and Polese et al. previously reported a strong positive correlation (r and R^2^ > 0.80) between spontaneous walking speed and energetic cost of walking (mL O_2_/kg/meter) after stroke and utilized this relationship to derive and validate equations that accurately predict the energy cost of walking during overground walking [60,61]. Moreover, walking speed has been used along with accelerometry data (i.e., distance walked) to estimate total energy expenditure (kcal) after stroke [59]. Importantly, one main difference between our work and that of Compagnat et al. and Polese et al. are the outcome measures used. Our work did not focus on estimating the energy cost of walking; rather, it focused on estimating the volume of oxygen consumed during walking regardless of distance and walking speed. Importantly, whereas VO_2_ is a measure of exercise intensity, the energy cost of walking is not. The volume of oxygen consumed during walking is only a component of the energy cost of walking and does not consider walking distance (i.e., mL O_2_/kg/min rather than mL O_2_/kg/m). This major difference between the previous and current research likely contributed to the contrasting findings of the significance of walking speed in the energy estimation equations. Moreover, comfortable walking speed may be more relevant in settings that emphasize overground walking, as these inherently introduce variability in speed, distance covered, and energy cost, making comfortable walking speed a potentially more influential factor. In contrast, our study’s use of treadmill-based exercise protocols and self-limited maximal effort testing to evaluate energy expenditure across a range of different intensities may have minimized the predictive contribution of comfortable walking speed. Indeed, treadmill walking may have reduced the variability in energy expenditure associated with different speeds and individual walking mechanics. Our findings may thus have limited generalizability to overground walking, where comfortable walking speed could play a more significant role. Future studies could investigate whether incorporating overground walking data or testing in mixed settings alters the contribution of comfortable walking speed to wVO_2_ estimation models.

Common methods for measuring exercise intensity in clinical and laboratory settings include the percentage of maximum oxygen uptake (VO_2_ max) [72], percentage of heart rate reserve (HRR) [73], rate of perceived exertion (RPE) [74], and metabolic equivalents (METs) [32]. One notable application of the wVO_2_ estimation equation in this study is to classify exercise intensity using METs, which were calculated from estimated wVO_2_. The performance of this classification approach was also improved by incorporating additional health-related and subject-specific variables, rather than relying solely on heart rate. For instance, in Figure 3, Subject 3 was classified as reaching vigorous exercise early, from stage 2, based on METs calculated using the wVO_2_ estimation equation with only heart rate (Equation (1)). However, this subject actually maintained moderate exercise intensity throughout the training period and never reached vigorous intensity, according to both the estimated METs from Equation (4) and actual METs. Additionally, Subject 7 was classified as entering vigorous intensity at stage 8 based on estimated METs from Equation (1). In contrast, estimated METs from Equation (4) and actual METs showed that this subject reached vigorous intensity at stages 4 and 5, respectively. These findings highlight the potential for overestimation or underestimation of METs and exercise intensity when relying solely upon heart rate-based wVO_2_ estimation. Such discrepancies could result in ineffective training or overexertion in real-world settings. This aligns with other research findings, which suggest that heart rate, when assessed alone, may not provide a complete picture of training intensity, particularly in post-stroke populations [75,76,77].

Our work may have been limited by the number of subjects included. The sample size is a fairly homogenous group of high-functioning, ambulatory, community-dwelling individuals with chronic stroke (7.39 ± 5.08 years) who were deemed medically appropriate to participate in a high-risk, maximal-effort exercise test or fast walking by their medical provider. Thus, our population sample does not likely account for the heterogeneous nature of post-stroke deficits and may not be capturing the significant variability in oxygen utilization that may exist across individuals who have experienced strokes of differing severity and/or experience different comorbidities. Future studies with larger and more heterogeneous samples (e.g., more acute strokes and additional pulmonary and/or cardiac diseases) could refine and potentially expand our estimation model, increasing its applicability to a broader range of individuals post stroke. Additionally, larger data sets could support subgroup analyses based on stroke characteristics and comorbidities, providing a more tailored approach to wVO_2_ estimation across the post-stroke population. This work is also limited by the variables that were available to be explored. For example, we did not have access to data measuring the uptake and utilization of oxygen by the muscles during walking, which is a significant contributor to wVO_2_ in both neurologically intact [78] and post-stroke individuals [5,79,80]. Future work could measure the uptake and utilization of oxygen by the muscles during walking directly or indirectly, or consider the inclusion of variables related to whole-body oxygen utilization and aerobic capacity that can be captured with a VO_2_ max test. Finally, our data collection was not blinded.

## 5. Conclusions

Compared to direct wVO_2_ measurements obtained by the gold-standard, indirect calorimetry, the wVO_2_ estimates predicted from heart rate data alone are highly inaccurate and result in the poor classification of exercise intensity. However, adding health-related clinical variables that are easy to collect at the point-of-care (i.e., BMI, age, and sex) to a heart rate-based estimation equation meaningfully enhances the accuracy and reliability of wVO_2_ estimations. Future research exploring the value of additional stroke-specific variables, such as measures of sensorimotor impairment or oxygen uptake and utilization by the muscles, may further improve wVO_2_ estimations.

## Figures and Tables

**Table 1:** General characteristics of subjects.

Subject	Age (y)	Chronicity (y)	Sex	BMI (kg/m^2^)	FGA
Cohort 1					
1	33	10.3	Male	26.68	28
2	55	7.1	Male	27.41	12
3	76	10.9	Male	30.95	18
4	62	11.5	Female	23.99	27
5	48	-	Female	21.96	-
6	56	13.5	Female	43.51	-
7	56	13.6	Female	21.32	24
8	49	16.9	Male	27.89	20
9	52	7.3	Female	18.55	-
10	62	2.6	Male	28.70	-
11	69	4.4	Male	26.56	22
Cohort 2					
12	61	3.0	Male	19.89	19
13	30	1.8	Male	28.41	14
14	55	1.4	Male	20.97	19
15	65	1.8	Male	24.47	15
16	61	4.8	Male	32.16	23

y—year; BMI—body mass index; kg/m^2^—kilogram per square meter; FGA—Functional Gait Assessment.

**Table 2:** Equation Development: Estimation equations predictor selection summary.

Step	Predictor Entered	Adj. R^2^	C(p)	AIC	SBIC	RMSE (mL O_2_/kg/min)	%∆ RMSE (%)	MAE (mL O_2_/kg/min)	%∆ MAE (%)
1	HR (“heart rate only” model)	0.53	1633	6530	6545	3.79	--	2.98	--
2	HR + BMI	0.70	623	6004	6024	3.03	−20.05	2.33	−21.81
3	HR + BMI + Age	0.76	233	5715	5740	2.68	−11.55	1.96	−15.88
* 4	HR + BMI + Age + Sex	0.80	29	5530	5561	2.48	−7.46	1.93	−1.53
5	HR + BMI + Age + Sex + CWS	0.80	6	5507	5543	2.45	−1.21	1.92	−0.52

Adj. R^2^—adjusted R-squared; C(p)—Mallow’s C(p); AIC—Akaike information criterion; SBIC—Sawa’s Bayesian information criterion; RMSE—root mean square error; mL O_2_/kg/min—milliliters of oxygen consumed per kilogram body weight per minute; %∆ RMSE—percent change in root mean square error; MAE—mean absolute error; %∆ MAE—percent change in mean absolute error; HR—heart rate; BMI—body mass index; CWS—comfortable walking speed during 10 m walk test; *—the step with the best overall fit and accuracy of estimation, along with meaningful improvement as defined.

**Table 3:** Equation Validation: Accuracy and reliability of equation-generated wVO_2_ estimations versus indirect calorimetry wVO_2._

Equation	Estimation Equation	R^2^	RMSE (mL O_2_/kg/min)	%∆ RMSE (%)	MAE (mL O_2_/kg/min)	%∆ MAE (%)	ICC
1	HR (“heart rate only”)	0.53	3.85	--	3.11	--	0.68
2	HR + BMI	0.69	3.09	−19.74	2.39	−23.15	0.81
3	HR + BMI + Age	0.76	2.76	−10.68	2.05	−14.23	0.86
* 4	HR + BMI + Age + Sex	0.79	2.55	−7.61	1.99	−2.93	0.88
5	HR + BMI + Age + Sex + CWS	0.79	2.56	0.39	2.03	2.01	0.88

R^2^—R-squared; RMSE—root mean square error; mL O_2_/kg/min—milliliters of oxygen consumed per kilogram body weight per minute; %∆ RMSE—percent change in root mean square error; MAE—mean absolute error; %∆ MAE—percent change in mean absolute error; ICC—intra-class correlation coefficient; HR—heart rate; BMI—body mass index; CWS—comfortable walking speed during 10 m walk test; *—the most accurate and reliable equation with meaningful improvement as defined.

## Data Availability

Data are available on request.

## Supplementary Materials

The following supporting information can be downloaded at: https://www.mdpi.com/article/10.3390/bioengineering11121250/s1, Table S1: wVO_2_ and resting VO_2_ baseline of subjects; Table S2: Estimation equation coefficients, Material S3: An Excel spreadsheet that can automatically calculate estimated wVO2, METs, and classify exercise intensity using the optimal estimation equation.
