# Supplementary material for: Enhancing Heart Rate-Based Estimation of Energy Expenditure and Exercise Intensity in Patients Post Stroke
Source: Bioengineering (Basel). 2024 Dec 10;11(12):1250. doi: 10.3390/bioengineering11121250 (PMC11673045; doi:10.3390/bioengineering11121250)
Supplement: Supplementary file 1 [file bioengineering-11-01250-s001.zip › bioengineering-3303097-supplementary-1205 1.pdf]

Supplementary Materials

Table S1. wVO_2 and resting VO_2 baseline of subjects

Subject	VO_2 at Rest (mL O_2/kg/min)	Exercise Test Stage*	Average of wVO_2 (mL O_2/kg/min)	SD of wVO_2 (mL O_2/kg/min)
**Cohort 1**				
1	4.1	1	19.0	4.3
		2	23.1	2.1
		3	24.9	1.8
		4	26.3	1.5
		5	28.7	2.5
		6	30.8	1.7
2	3.0	1	15.2	1.6
		2	15.7	1.4
		3	18.6	1.1
		4	22.5	1.5
3	4.7	1	13.3	2.2
		2	15.1	1.7
		3	17.1	1.7
		4	18.0	2.6
		5	18.6	1.7
		6	20.4	1.9
4	3.9	1	22.5	2.1
		2	24.0	3.2
		3	29.2	3.1
		4	30.7	2.7
		5	30.8	4.5
		6	32.2	3.5
5	5.6	1	19.1	3.1
		2	22.8	3.2
		3	26.2	4.4
		4	30.3	2.7
		5	35.0	6.0
		6	34.9	2.9
		7	31.8	2.9
		8	31.5	3.1
6	5.9	1	12.4	1.8
		2	15.5	0.6
7	5.3	1	14.8	3.0
		2	17.0	1.9
		3	18.4	2.3
		4	20.4	2.6
		5	21.6	1.6
		6	22.7	2.1
		7	23.4	2.0
		8	24.7	1.6
		9	25.1	2.2
8	2.2	1	11.2	1.6
		2	12.1	2.0
		3	16.1	2.0

		4	16.6	2.1
		5	19.9	1.4
		6	21.7	1.8
9	4.2	1	13.5	1.9
		2	14.3	1.9
		3	15.3	1.3
		4	17.6	2.2
		5	17.9	1.0
		6	18.3	2.3
10	2.3	1	8.2	1.2
		2	9.5	1.9
		3	9.5	2.4
		4	9.2	1.6
		5	10.4	2.7
11	3.3	1	10.9	1.2
		2	11.4	2.1
		3	12.8	1.4
		4	15.2	1.7
**Cohort 2**				
12	3.7	SS	11.5	0.7
		CWS	12.1	1.4
		Fast 1	14.9	1.0
		Fast 2	15.4	0.4
13	4.7	SS	13.5	1.6
		CWS	16.4	0.7
		Fast 1	18.3	1.1
		Fast 2	21.0	1.5
14	4.0	SS	15.5	0.4
		CWS	15.9	0.4
		Fast 1	19.0	0.3
		Fast 2	24.4	1.9
15	3.3	SS	12.5	1.7
		CWS	14.2	0.5
		Fast 1	16.6	0.7
		Fast 2	18.6	1.1
16	4.1	SS	13.4	0.6
		CWS	16.2	0.7
		Fast 1	19.1	0.5
		Fast 2	21.1	0.4

mL O_2/kg/min – milliliters of oxygen consumed per kilogram body weight per minute; SD – standard deviation; SS – slow speed; CWS – comfortable walking speed during 10 m walk test; Fast 1 – fast 1 speed; Fast 2 – fast 2 speed

* Each stage of the exercise tests lasts about 2 minutes

Table S2. Estimation equation coefficients

Equation	Equation predictors	β_0	β_1	β_2	β_3	β_4	β_5
1	HR (“heart rate only” model)	-4.84	0.20	--	--	--	--
2	HR + BMI	10.83	0.22	-0.68	--	--	--
3	HR + BMI + Age	16.95	0.21	-0.59	-0.13	--	--
4	HR + BMI + Age + Sex	5.09	0.19	-0.02	-0.18	4.53	--
5	HR + BMI + Age + Sex + CWS	3.90	0.19	-0.04	-0.16	4.53	1.71

β - beta coefficient; HR – heart rate; BMI – body mass index; CWS – comfortable walking speed during 10 m walk test

Material S3. An Excel spreadsheet that can automatically calculate estimated wVO_2, METs, and classify exercise intensity using the optimal estimation equation. * Please see the single Excel attachment.
